# Structural Basis for Importin-α Binding of the Human Immunodeficiency Virus Tat

**DOI:** 10.1038/s41598-017-01853-7

**Published:** 2017-05-10

**Authors:** K. M. Smith, Z. Himiari, S. Tsimbalyuk, J. K. Forwood

**Affiliations:** Charles Sturt University, School of Biomedical Sciences, Wagga Wagga, 2678 Australia

## Abstract

HIV-1 has caused 35 million deaths globally, and approximately the same number is currently living with HIV-1. The trans-activator of transcription (Tat) protein of HIV-1 plays an important regulatory function in the virus life cycle, responsible for regulating the reverse transcription of the viral genome RNA. Tat is found in the nucleus of infected cells, but can also invade uninfected neighbouring cells. Regions within Tat responsible for these cellular localisations are overlapping and include a nuclear localisation signal (NLS) spanning ^48^GRKKRR, and a cell penetrating peptide (CPP) signal spanning ^48^GRKKRRQRRRAPQN. However, the mechanism by which this NLS/CPP region mediates interaction with the nuclear import receptors remains to be resolved structurally. Here, we establish that the HIV-1 Tat:NLS/CPP is able to form a stable and direct interaction with the classical nuclear import receptor importin-α and using x-ray crystallography, we have determined the molecular interface and binding determinants to a resolution of 2.0 Å. We show for the first time that the interface is the same as host factors such as Ku70 and Ku80, rather than other virus proteins such as Ebola VP24 that bind on the outer surface of importin-α.

## Introduction

The HIV-1 virus has spread worldwide, infecting 60 million people, and causing more than 25 million deaths. More than 30 million people currently live with the disease^1^, but despite highly active antiretroviral therapy (HAART) reducing the effects of the virus, these antivirals do not clear the virus from infected patients. HIV-1 encodes three groups of proteins that are common in all retroviruses. The gag polyprotein, pol polyprotein and gp160 precursors are structural proteins that form the outer shell of the virus particle, and are processed to produce proteins for the virion interior. The accessory regulatory proteins, Vif, Vpr, Vpu and Nef, interact with cellular ligands and function as adapter molecules or to inhibit normal host function. The third group are the essential regulatory elements, Tat and Rev. The primary role of Tat is in regulating the reverse transcription of viral genome RNA, whilst Rev is responsible for the synthesis of major viral proteins for viral replication^2^.

Tat is a transcriptional trans-activator and plays an important role during HIV-1 replication by binding to a short-stem loop structure, known as the transactivation response element (TAR) located at the 5′ end of HIV RNAs. It assists in the elongation phase of HIV-1 transcription so that full-length transcripts can be produced^3^, and these functions occur within the nucleus of infected cells. Tat has been shown to localise to the nucleus in many studies, however, the mechanism by which it interacts with the nuclear import receptors has not been elucidated structurally^4, 5^.

Nuclear import can occur through passive diffusion (<45 kDa) or by energy dependent nuclear import receptors. The classical nuclear import pathway is the best characterised mechanism and is mediated by an adaptor molecule, importin-α, also known as the classical nuclear import receptor, binding cargo that can display a nuclear localisation signal (NLS). The transport carrier importin-β interacts with importin-α, and mediates translocation across the nuclear envelope through interactions with the nucleoporin proteins lining the nuclear pore complex^6, 7^. Upon entry to the nucleus, the heterotrimer transport complex is dissociated by the small GTPase Ran, releasing the NLS-containing cargo, and allowing recycling of the import receptors back to the cytoplasm^8, 9^.

The HIV-1 Tat derived cell penetrating peptide (^48^GRKKRRQRRRAPQN^61^;CPP) has been shown to effectively carry a large range of cargoes, from nanoparticles, peptides, nucleic acids and even proteins into cells and the nucleus^10–14^. *In vitro* studies have shown that Tat is able to bind nuclear import receptors which mediate nuclear localisation^5, 15^, however, a structural basis for this interaction remains to be elucidated. There has also been some debate in the literature about whether Tat can bind directly to importin-α^16^ or importin-β^15^. To determine the precise binding determinants that mediate interaction between the nuclear import receptor and Tat, the entire cell penetrating region of HIV-1 Tat, ^48^GRKKRRQRRRAPQN^61^, was recombinantly expressed as a GST-fusion and tested for binding to both importin-α and importin-β^6, 16^. We found a strong and direct interaction between Tat:NLS/CPP and importin-α, and no direct interaction with importin-β. Together with structural elucidation of the interface by x-ray crystallography, this study provides new insights into the interface between these two proteins which mediate localisation of Tat to the nucleus.

## Materials and Methods

### Plasmid preparation

Tat residues (^48^GRKKRRQRRRAPQN^61^) were codon optimised for expression in *E. coli* and cloned into the PGEX4T-1 vector at BamHI/EcoRI sites with an additionally engineered N-terminal TEV site for GST-tag cleavage. An isolate of mouse importin-α (homologue of human importin-α; 95% sequence identity) that lacks the auto-inhibitory N-terminal importin-β binding (IBB) domain (residues 70–529) and cloned into the pET30 expression vector has been described previously^17^. An isolate of mouse importin-β (KPNB1, homologue of human importin-α: 99% sequence identity) was cloned into the pMCSG21 vector using protocols described previously^18, 19^.

### Recombinant Expression and Purification

Overexpression of importin-α and importin-β was performed using the autoinduction method according to Studier^20^ and purified as outlined previously^21^. Briefly, cells were resuspended in His buffer A (50 mM phosphate buffer, 300 mM NaCl, 20 mM Imidazole, pH 8), and lysed by two freeze-thaw cycles. The soluble cell extract was injected onto a five mL HisTrap HP column (GE Healthcare) and washed with twenty column volumes of His buffer A on an AKTApurifier FPLC. The sample was eluted using an increasing concentration gradient of imidazole, and eluent fractions were pooled and loaded onto a HiLoad 26/60 Superdex 200 column, pre-equilibrated in buffer A (50 mM Tris pH 8, 125 mM NaCl). Fractions corresponding to the correct molecular weight were collected, and assessed for purity by SDS-PAGE.

Tat-NLS was over-expressed as a GST-fusion protein using IPTG to induce the cells at an OD_600_ of 0.6. Following growth for 24 h, cells were harvested by centrifugation, at 6,000 rpm for 30 min and the cell pellet resuspended in buffer A and stored at −20 °C. The soluble cell extract containing the GST:Tat-NLS fusion was injected onto a GST 5 mL HP column pre-equilibrated in GST buffer A and washed with GST buffer A until the UV reading stabilised to baseline. The purified importin-α or importin-β was then added to the GST column, washed for a further ten column volumes, and eluted in GST buffer A containing 10 mM glutathione. To cleave the GST affinity tag from the Tat:NLS/CPP importin-α, TEV protease cleavage was undertaken overnight, and continued until cleavage was complete. The protein mixture was further purification by size-exclusion chromatography on a HiLoad 26/60 Superdex 200 column (GE Healthcare), and the complex concentrated to 7 mg/mL using an Amicon MWCO 10 kDa filter, aliquoted, and stored at −80 °C.

### Crystallization of Tat-NLS with Importin-α

The final crystallisation condition contained 1.25 M sodium citrate pH 7 and 10 mM DTT. The Tat:NLS/CPP importin-α complex was screened using hanging drop vapour diffusion method where the protein was mixed in a 1:1 ratio with crystallization condition containing reservoir solution and incubated at 296 K. Large rod shaped crystals grew after four days, and the crystals were cryoprotected in a reservoir solution 20% glycerol prior to being flash cooled to 100 K in liquid nitrogen.

### Data Collection and Structure Determination

A single crystal was used to collect x-ray diffraction data on the MX2 crystallography beamline at the Australian Synchrotron. The diffraction data was processed using iMosflm^22^ and scaled and merged using Aimless^23, 24^. The data was phased by molecular replacement using Phaser^25^ and Protein Data Bank (PDB) structure 5FC8 as a search model. Model rebuilding and refinement were undertaken in Coot^26–28^ and Phenix^29^, respectively.

### GST-pulldown Assay for Affinity Determination

For determination of the dissociation constant, K_D_, glutathione agarose beads washed in GST buffer A were saturated with purified GST-Tat:NLS/CPP. Beads were washed three times in GST buffer A and distributed equally in 10 µL aliquots. To each aliquot, 100 µL of two-fold serially diluted importin-α was added, with the initial concentration of 30 µM. One additional tube containing 0 µM importin-α was used as a control. The samples were incubated for one hour at 4 °C before being washed twice with 1 mL of GST buffer A. Binding was assessed by adding 20 µL of tris-glycine sample loading buffer to the beads, boiled for 10 min, and analysed by SDS-PAGE. Images were recorded using BioRad Gel Doc system and processed in ImageJ^30^. The data was normalised across each replicate experiment and data analysed using one-site specific binding analysis performed in Prism version 7.0b for Mac, GraphPad Software, La Jolla California USA, www.graphpad.com.

## Results

### The Tat:NLS/CPP region forms a direct interaction with importin-α

**Figure 1:**
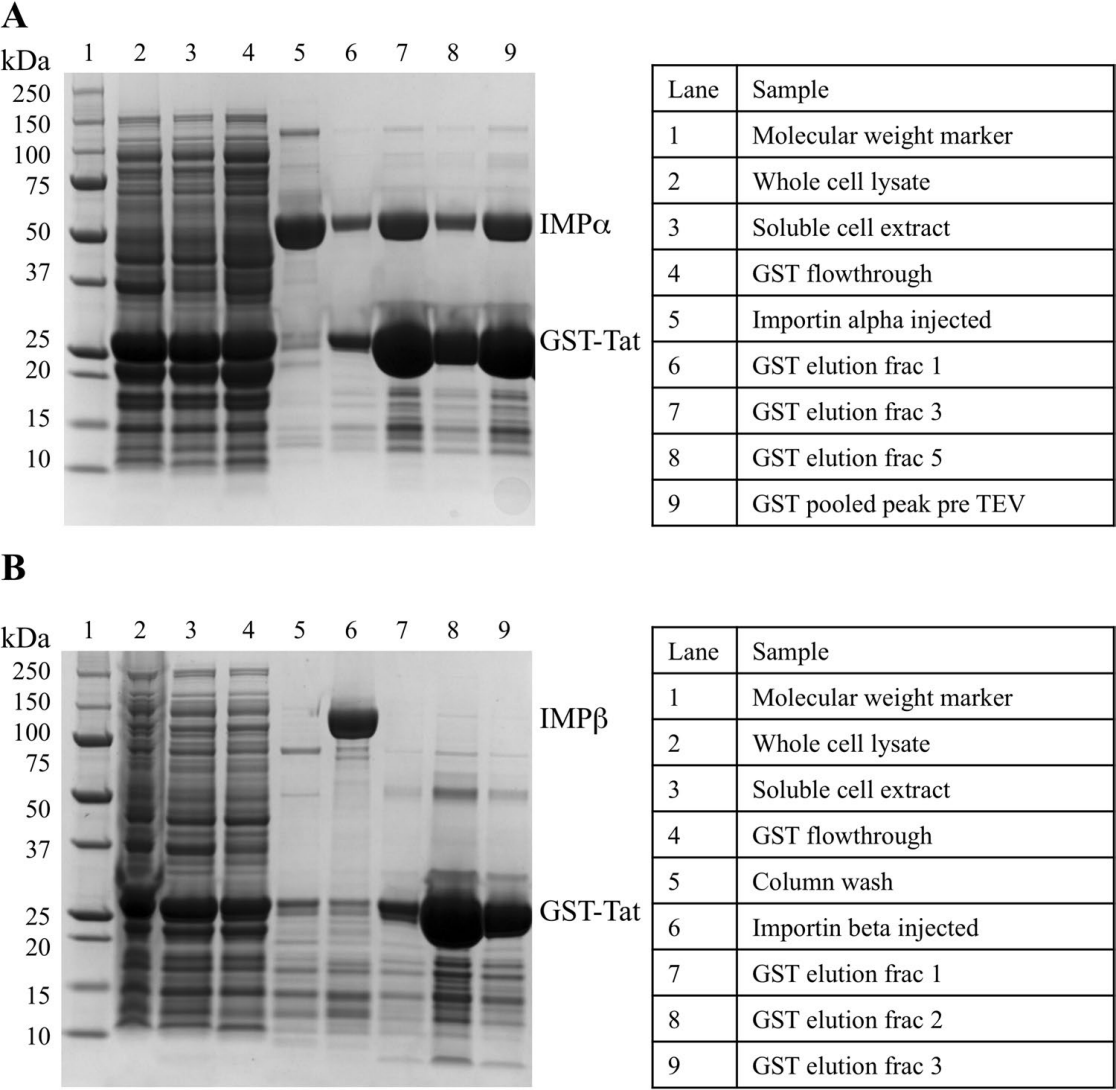
Binding of Tat:NLS/CPP to importin-α and importin-β. (**A**) SDS-PAGE visualization of complex formation between Tat:NLS/CPP and importin-α. (**B**) SDS-PAGE revealing a lack of complex formation between Tat:NLS/CPP and importin-β. Both gels were cropped at the right to remove samples from additional purification steps and other experiments. The full gels are presented in the Supplementary Figure 1.

The NLS/CPP region of Tat, spanning residues 49–61, have been shown to contain a functional NLS, however, there has been recent debate as to whether the highly basic cell penetrating peptide region is bound using the importin-α adapter, or can bind directly to importin-β. Since this region contains a large stretch of positively charged residues, many of which of which could fit the definition of a classical NLS binding to importin-α, or an Arg rich importin-β interaction, we tested binding against both types of receptors. Here, we immobilised the GST-Tat:NLS/CPP fusion protein onto a glutathione column, washed the column, then passed each respective importin over the immobilised proteins to assess binding. We observed that most of the importin-α was retained on the column (Fig. 1A), whilst little, if any importin-β remained bound (Fig. 1B). These results indicate a direct binding between the Tat:NLS/CPP and the classical nuclear import receptor importin-α.

### Protein purification and complex formation

To determine the structural basis for the interaction between the nuclear import receptor importin-α and Tat NLS/CPP, both proteins were purified to homogeneity and isolated as an equimolar complex using the following series of purifications. The nuclear import receptor importin-α was first purified by 6-His affinity and size exclusion chromatography, then loaded on a column containing purified GST-Tat:NLS/CPP. The excess importin-α was removed by washing the column extensively and following elution, the GST affinity tag was removed by proteolytic cleavage with the TEV protease. The mixture was then purified by size exclusion chromatography, where the importin-α:Tat NLS/CPP complex (>58 kDa) was successfully separated from excess Tat NLS/CPP (<5 kDa), resulting in a homogenous equimolar complex for crystallisation.

### Protein crystallisation and data collection

**Figure 2:**
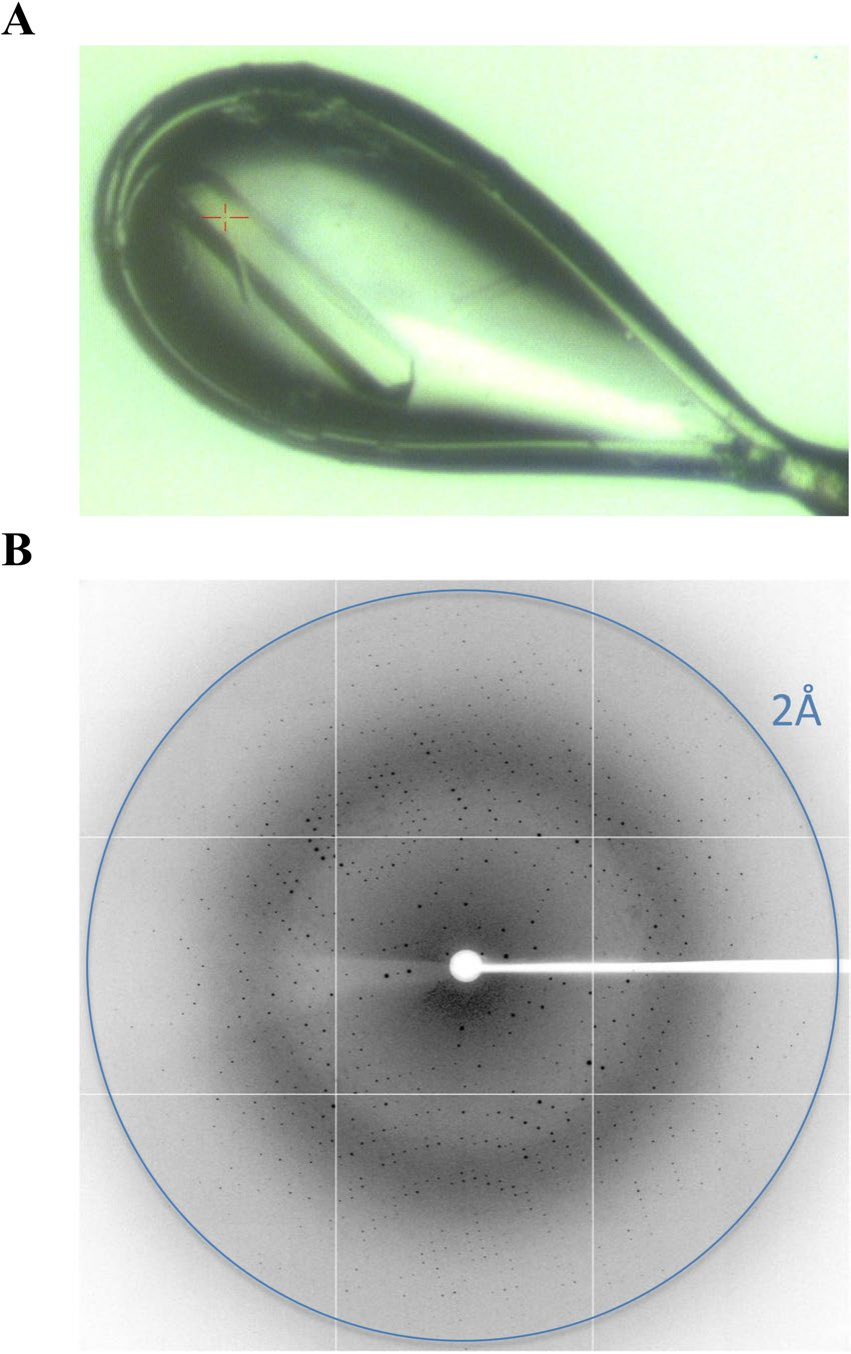
Tat:NLS/CPP importin-α crystal diffraction. (**A**) Rod-shaped crystal of Tat:NLS/CPP importin-α shown used for data collection (**B**) X-ray diffraction image obtained from the Tat:NLS/CPP importin-α crystal at the Australian Synchrotron.

The hanging-drop vapour diffusion method was used to obtain large rod-shaped crystals after four days (Fig. 2A). The crystal diffracted to 2.0 Å (Fig. 2B) resolution on the MX2 beam line at the Australian Synchrotron, and a total of 110**°** of data, collected at 0.5° oscillations, were indexed, merged and scaled using *iMosflm* v.1.0.7^22^ and AIMLESS^23, 24^ (Table 1). The structure was solved by molecular replacement using Phaser^25, 31^ with PDB 5FC8 as a model. Following rebuilding and refinement, the 2.0 Å resolution structure was refined to a R_work_/R_free_ of 0.16/0.19 using iterative cycles of refinement and modelling in Phenix^29^ and COOT^28^ respectively. The final model consisted of 426 residues of importin-α, 8 residues of the Tat:NLS/CPP, and 319 water molecules. The N- and C-terminal amino acid residues of the Tat:NLS/CPP peptide displayed poor density due to flexibility, allowing only accurate placement of the protein peptide chain. Stereochemistry and other refinement statistics are presented in Table 1.

**Table 1:** Data collection and refinement statistics for structure of importin-α in complex with HIV-1 Tat:NLS/CPP domain.

	Tat:NLS/CPP importin-α (PDB code 5SVZ)
Wavelength (Å)	0.9537
Data-collection temperature (K)	100
Detector Type	CCD
Detector	ADSC QUANTUM 315r
Mosaicity (°)	0.29
Resolution range (Å)	29.66–2.00 (2.05–2.00)
Space group	P 2_1_ 2_1_ 2_1_
Unit cell (Å)	79.00, 89.83, 99.46
Total reflections	212,694 (15,721)
Unique reflections	46,564 (3,439)
Multiplicity	4.6 (4.6)
Completeness (%)	96.5 (98.2)
Mean I/σ (I)	13.4 (2.4)
Wilson B-factor Å^2^	27.43
Matthew’s Coefficient (A^3^/Da)	3.09
Solvent content (%)	60.22
R_pim_	0.047 (0.52)
R_work_	0.17 (0.25)
R_free_	0.19 (0.27)
No. of non-hydrogen atoms	3633
Macromolecules	3319
Solvent	314
Protein residues	434
r.m.s.d.	
bond length (Å)	0.003
bond angle (°)	0.65
Ramachandran favored (%)	98
Ramachandran allowed (%)	1.6
Ramachandran outliers (%)	0

Values in brackets describe the highest resolution shell.

### Binding determinants of the HIV-1 Tat:NLS/CPP in complex with importin-α

**Figure 3:**
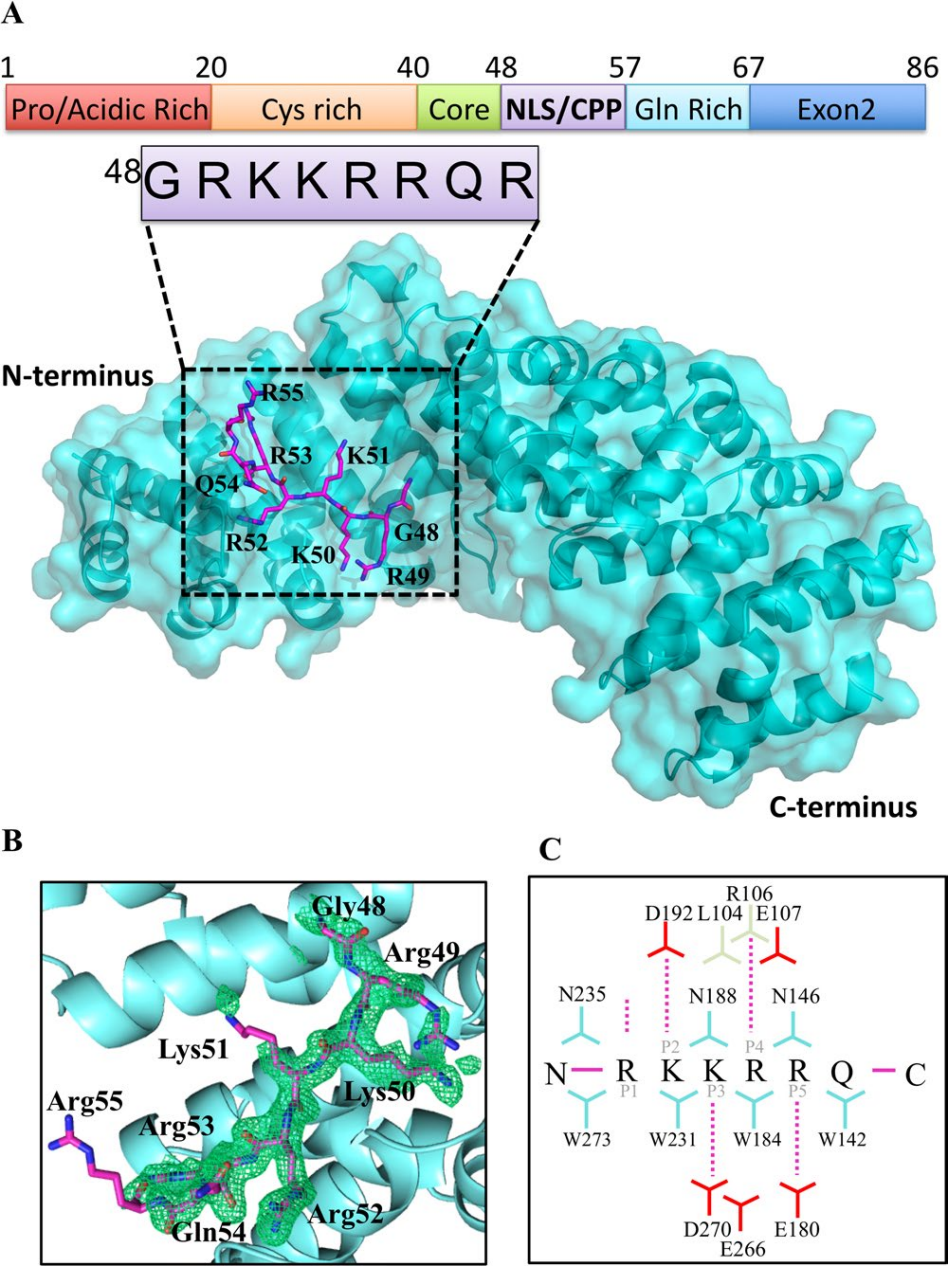
Crystal structure of Tat:NLS/CPP importin-α. (**A**) Full structure of Tat-NLS/CPP (purple sticks) and importin-α (cyan ribbons/transparent surface) complex. (**B**) Simulated annealing omit map (green mesh) of Tat-NLS/CPP shown at 3σ. (**C**) Schematic representation of importin-α Tat:NLS/CPP interactions. The NLS backbone is indicated as a horizontal magenta line, from the N- to the C-terminus. NLS side chains are represented as vertical dotted magenta lines. Selected importin-α Trp and Asn residues are shown in blue. Selected importin-α Asp and Glu residues are shown in red. Single amino acid code is used. Structure has been deposited to the PDB and issued the code 5SVZ.

**Figure 4:**
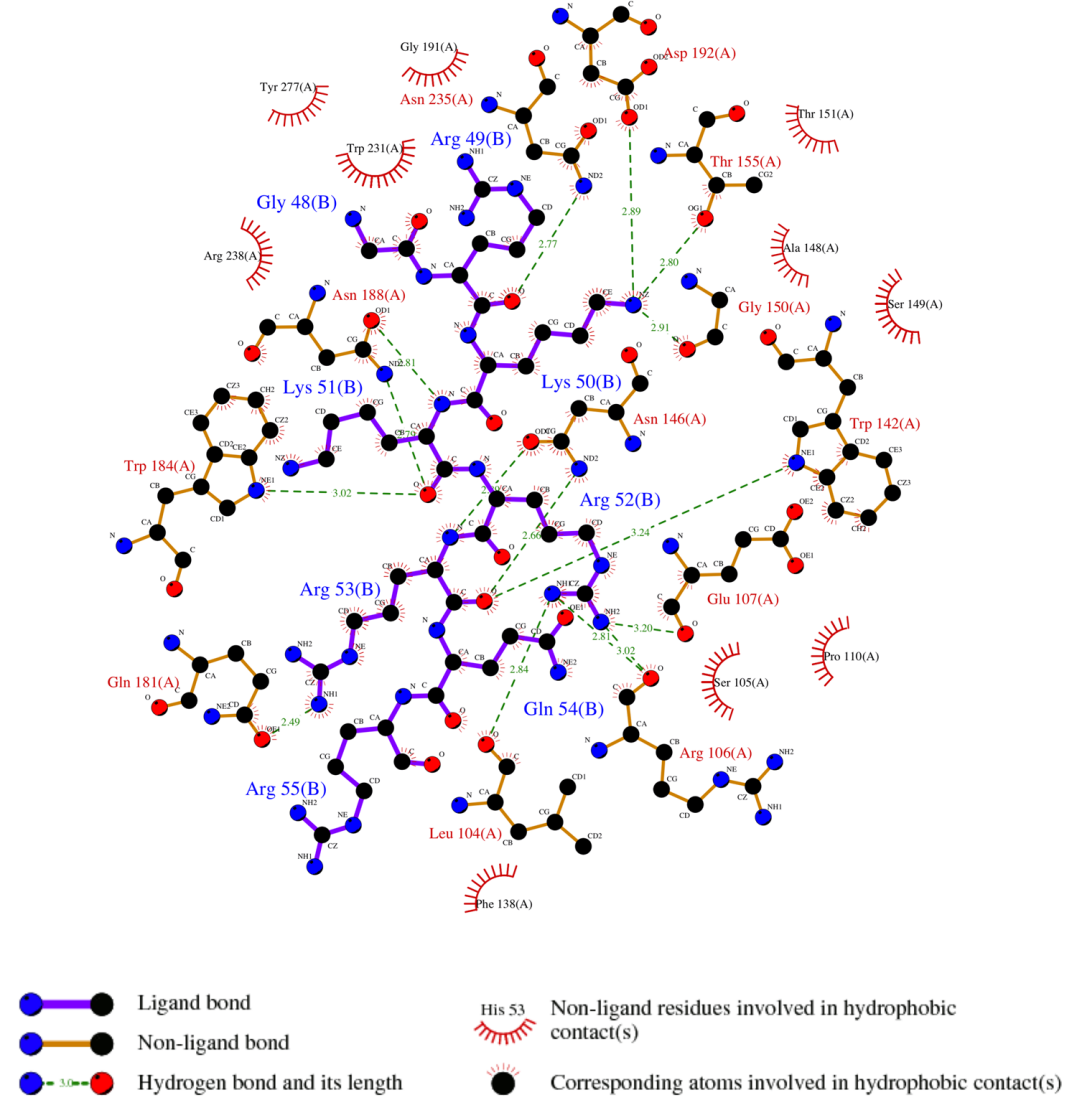
LigPlot interaction schematic for Tat:NLS/CPP importin-α structure. Tat:NLS/CPP intramolecular bonds are shown as solid purple lines, importin-α intramolecular bonds are shown as solid orange lines. Importin-α residues involved in hydrophobic contacts are shown as multiple red fanning lines. Corresponding atoms involved in hydrophobic contacts are shown with small orange fanning lines. Hydrogen bonds and their length are shown as green dotted lines. Figure was made using LigPlot^34^.

**Figure 5:**
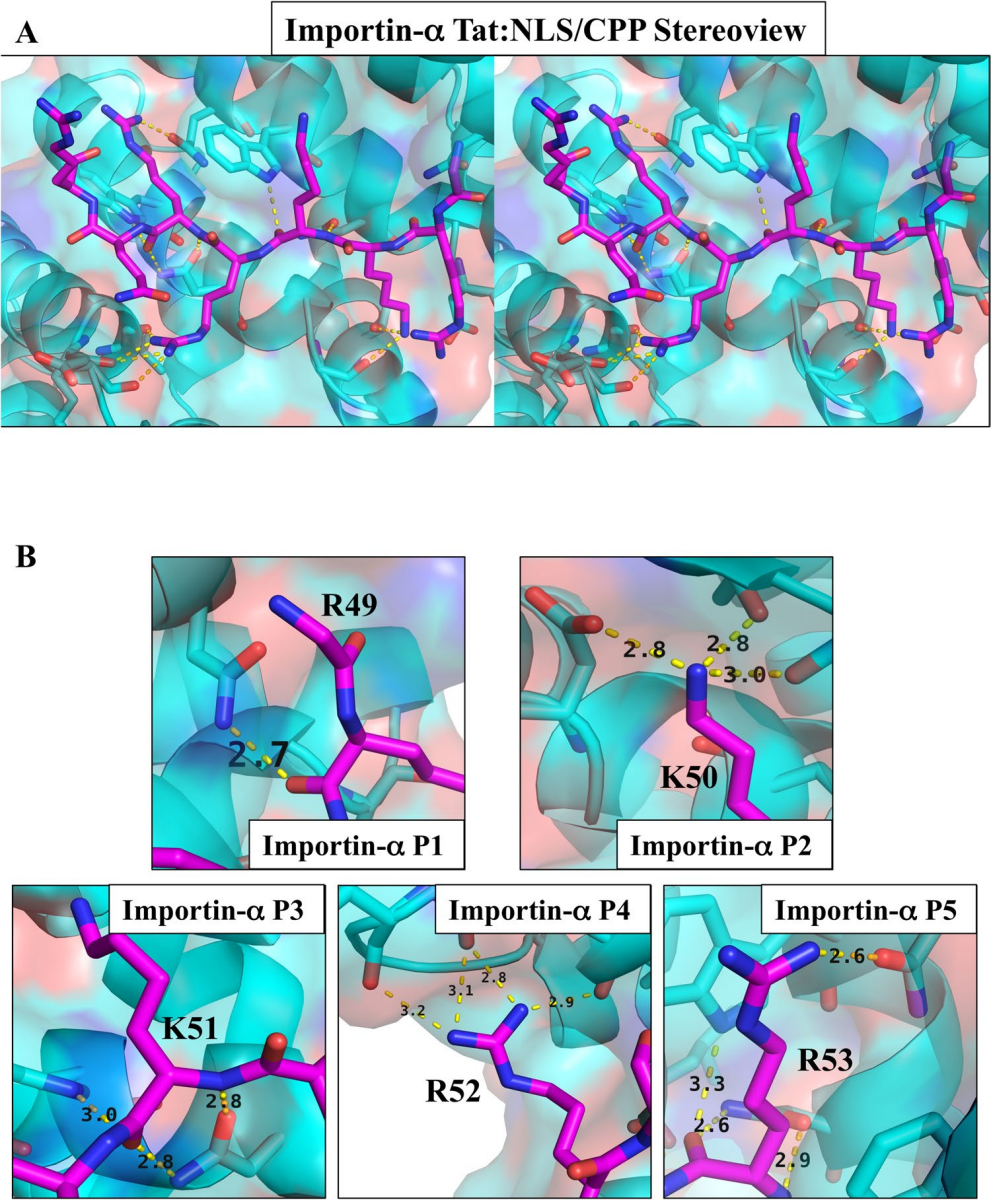
Importin-α interacting residues from Tat NLS/CPP region. (**A**) Importin-α shown in cyan (ribbons and surface view), Tat:NLS/CPP shown in magenta (sticks). NLS residues visible within crystal structure, and bound to the N-terminal concave ARM domains of importin-α, are shown in stereoview for 3D visualisation (**B**) Each P-site that interacts with importin-α is displayed with each interaction highlighted with dashed lines.

The overall structure of importin-α exhibited an all α-helical structure arranged as ten sequential armadillo (ARM) motifs as described previously^6^. The Tat:NLS/CPP binds to the major binding site of importin-α within ARM domains 2–4 (Fig. 3A,B). There are no NLS residues in the minor binding site, indicating that the NLS region is monopartite. The main chain of Tat residue Arg49 interacts with the side chain of importin-α residue Asn235 in the P1 site (Figs 4 and 5). Tat residue Lys50 binds importin-α at the P2 site where it forms a salt bridge with importin-α residue Asp192 as well as additional side chain interactions with importin-α residues Gly150 and Thr155. The Tat peptide backbone of residue Lys51 hydrogen bonds the side chains of importin-α residues Asn188 and Trp184 in the importin-α P3 binding site. The side-chain of Tat residue Arg52 in the P4 position hydrogen bonds with the importin-α main chain of residues Leu104, Arg106, and Glu107. The P5 binding site is occupied by Tat residue Arg53 which makes main chain interactions with the side chains of importin-α residues Trp142 and Asn146. The overall binding buries 717 Å^2^ of surface area, and is mediated by 15 hydrogen bonds and 1 salt bridge interaction (Figs 3 and 4). Further details on the NLS binding determinants are shown in Figs 4B and 5 and summarised in Table 2.

**Table 2:** Tat:NLS/CPP hydrogen bond interactions with importin-α generated by PDBSum^35–38^ *Salt bridge interaction.

Tat:NLS/CPP				Importin-α		
Atom	Residue	Number	Distance (${\rm{\AA }}$)	Atom	Residue	Number
NH1	Arg	52	2.84	O	Leu	104
NH1	Arg	52	2.81	O	Arg	106
NH2	Arg	52	3.02	O	Arg	106
NH2	Arg	52	3.20	O	Glu	107
O	Arg	53	3.24	NE1	Trp	142
N	Arg	53	2.89	OD1	Asn	146
O	Arg	53	2.66	ND2	Asn	146
NZ	Lys	50	2.91	O	Gly	150
NZ	Lys	50	2.80	OG1	Thr	155
NH1	Arg	53	2.49	OE1	Gln	181
O	Lys	51	3.02	NE1	Trp	184
N	Lys	51	2.81	OD1	Asn	188
O	Lys	51	2.79	ND2	Asn	188
*NZ	Lys	50	2.89	OD1	Asp	192
O	Arg	49	2.77	ND2	Asn	235

### Binding affinity of the HIV-1 Tat:NLS/CPP in complex with importin-α

**Figure 6:**
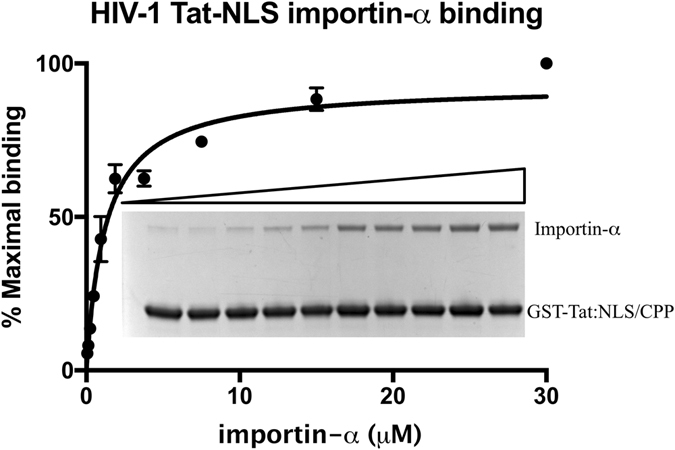
Quantitative GST-pull down for binding affinity determination. Glutathione agarose containing the GST-Tat:NLS/CPP incubated and washed with two-fold serially diluted importin-α (initial concentration of 30 µM). Samples analysed by SDS-PAGE and images recorded using BioRad Gel Doc system were processed from triplicate gels and processed in ImageJ and analysed using one-site specific binding in Prism 7.0. A representative gel showing binding is included, and the original, uncropped gel is provided in Supplementary Figure 2.

To estimate the binding affinity, importin-α was serially titrated against equal concentrations of HIV-1 Tat:NLS/CPP, and binding captured using a GST-pulldown. The binding affinity was determined to be 1.2 ± 0.2 µM from three replicates (Fig. 6). The binding affinity measured for HIV-1 Tat:NLS/CPP is in the low micromolar range and similar to previously reported values of other NLSs including Dengue 2 C-terminal NS5, 0.27 ± 0.1 µM; and Dengue 3 C-terminal NS5 0.37 ± 0.11 µM^32^.

## Discussion

**Figure 7:**
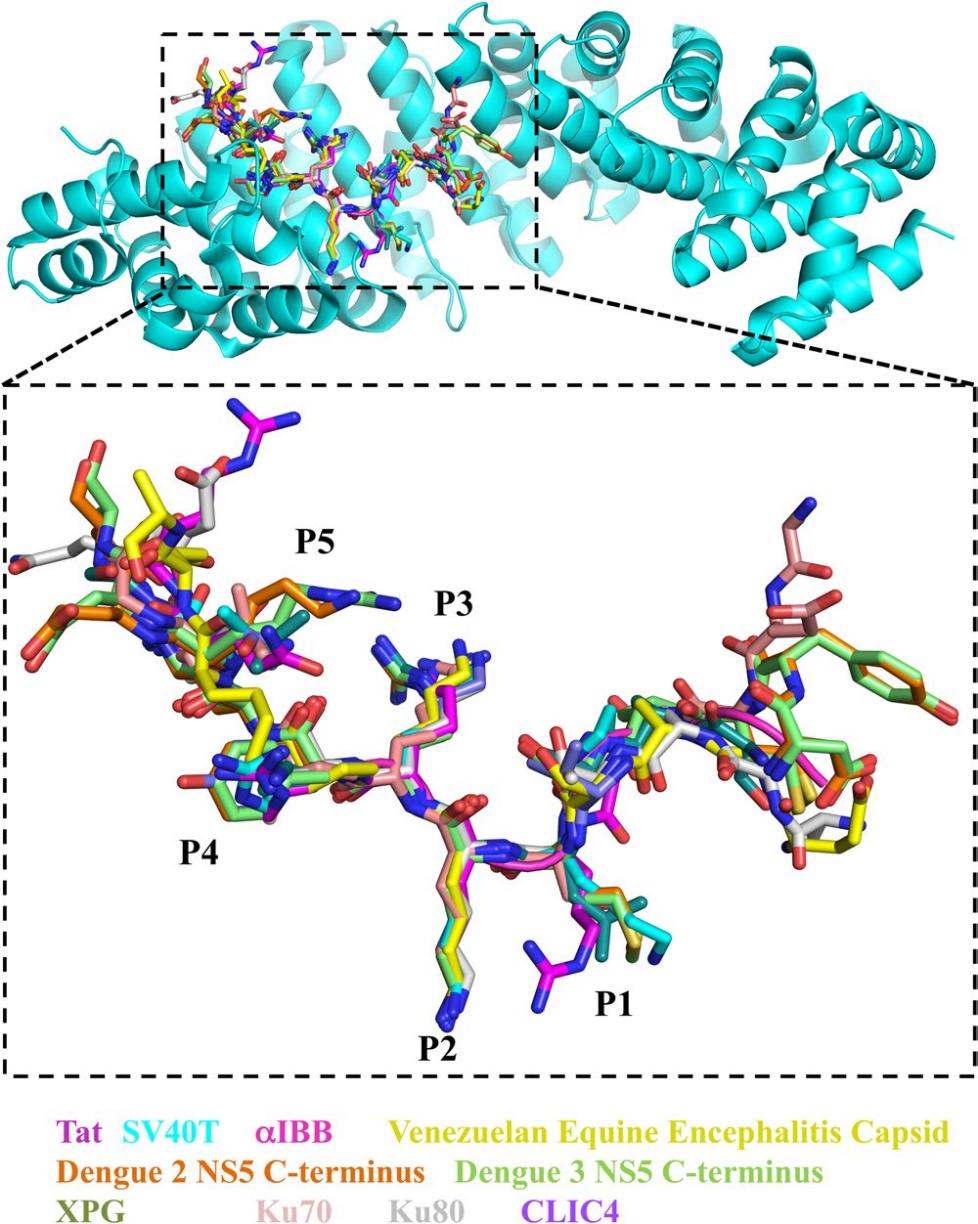
Overlay of cNLSs bound at the major site of importin-α. Conservation of NLS structures are coloured accordingly: Tat (magenta), SV40T (cyan), αIBB (pink), Venezeualan equine encephalitis virus (gold), Dengue 2 NS5 C-terminus (orange), Dengue 3 NS5 C-terminus (green), XPG (dark green), Ku70 (nude), Ku80 (grey), CLIC4 (purple), all overlaid onto Tat:NLS/CPP bound importin-α (cyan). Overlay of NLSs are enlarged within the P1-P5 positions.

There has been contention as to which nuclear import receptor is responsible for the nuclear translocation of Tat. One study suggests Tat is importin-α mediated^15^, whereas another study has shown that it is dependent on importin-β^16^. Here, we show that the C-terminal ^55^RRR is not providing additional binding to importin-α, and of the residues visible in the crystal structure ^48^GRKKRRQR, only residues ^48^GRKKRR mediate binding with importin-α. Our results support the findings of Ruben *et al*., who have shown nuclear import can be mediated by Tat-NLS/CPP residues GRKKR^16^. This binding motif is consistent with the previously defined monopartite class 2 NLSs^33^. The P1-P5 binding determinants in Tat:NLS/CPP are resolved within the presented 2.0 Å structure, and are consistent with previously solved structures (Table 3 and Fig. 7).

**Table 3:** PDB deposited cNLSs binding to the major site of importin-α.

NLS	Importin-α major binding site					PDB ID
	*P1*	*P2*	*P3*	*P4*	*P5*	
HIV1 Tat	R	K	K	R	R	This study
SV40T^7, 39, 40^	K	K	K	R	K	1EJL, 1BK6, 1Q1S, 1Q1T
αIBB^6^	L	K	K	R	N	1IAL, 1IQ1
Venezuelan Equine Encephalitis Capsid	A	K	K	P	K	3VE6
Ku70^41^	S	K	R	P	K	3RZX
Ku80^41^	A	K	K	L	K	3RZ9
CLIC4^42^	A	K	K	Y	R	3OQS
Dengue 2 NS5 C-terminus	M	K	R	F	R	5FC8
Dengue 3 NS5 C-terminus	M	K	R	F	R	5HHG
XPG^43^	S	K	R	K	R	5EKF, 5EKG

The major binding site of importin-α interact with five principal binding determinants in NLSs, known as P1-P5 (Table 3). These binding determinants P1-P5 bind between ARM domains 2–4 in importin-α and are conserved across previously determined NLS structures (Fig. 7). Position 1 (P1) on an NLS is the most divergent as only a main chain interaction is made with importin- α. Position 2 (P2) is the most highly conserved with a strict requirement of a lysine residue at this position to form the critical salt bridge interaction with importin-α residue D192 as well as side chain interactions with the main chains of importin-α residues T155 and G150. Position 3 (P3), position 4 (P4), and position 5 (P5), prefer long basic amino acids, however, for all characterised NLSs, there is a great variability in the amino acids at these positions (Fig. 7). The P3 site which is less conserved than the P2 site, can bind to Lys, Arg, and Leu residues, whilst the P5 site is similarly conserved and can bind Lys, Arg, and Asn residues. The P4 site is less conserved than P4 and P5, and can bind Pro, Phe, Arg, Lys, Leu, Tyr, and P1 is the least conserved binding to Met, Asn, Lys, Arg, Leu, Ala, and Ser residues^32^ (Table 3). The Tat:NLS/CPP (Fig. 5A,B) residues ^49^RKKRR fit within the requirements of the P1-P5 NLSs and bind importin-α in a similar fashion to other importin-α bound NLS structures, whilst other positively charged residues ^55^RRR were found not to bind in our structure. The similarities between HIV-1 Tat:NLS/CPP and previously reported NLS importin-α structures can be seen in sequence conservation (Table 2), structural overlay comparisons (Fig. 7) and from calculated RMSD values that varied from 0.150–0.431 Å (Table 4). Overall, this is the first time the NLS region within HIV-1 Tat has been determined structurally by X-ray crystallography. Through interaction with the N-terminal arginine rich motifs in importin-α, eight amino acids in Tat have been revealed and binding determinants identified that mediate interaction with the classical nuclear import receptor.

**Table 4:** RMSD differences of PDB deposited cNLSs binding to the major site of importin-α.

NLS	PDB	RMSD for Cα residues to Tat (${\rm{\AA }}$)
SV40T^7, 39, 40^	1EJL	0.217
αIBB^6^	1IAL	0.206
Venezuelan Equine Encephalitis Capsid	3VE6	0.315
Ku70^41^	3RZX	0.274
Ku80^41^	3RZ9	0.431
CLIC4^42^	3OQS	0.154
Dengue 2 NS5 C-terminus	5FC8	0.219
Dengue 3 NS5 C-terminus	5HHG	0.350
XPG^43^	5EKF	0.150

## Electronic supplementary material

Supplementary Information
