# Supplementary material for: Structural Basis for Importin-α Binding of the Human Immunodeficiency Virus Tat
Source: Sci Rep. 2017 May 10;7:1650. doi: 10.1038/s41598-017-01853-7 (PMC5431807; doi:10.1038/s41598-017-01853-7)
Supplement: Supplementary file 1 — Supplementary Information [file 41598_2017_1853_MOESM1_ESM.pdf]

Supplementary Information

Structural Basis for Importin-α Binding of the Human Immunodeficiency Virus Tat

K.M. Smith[1#], Z. Himiari[1#], S. Tsimbalyuk[1], J.K. Forwood[1*].

[1]Charles Sturt University, School of Biomedical Sciences, Wagga Wagga, 2678, Australia.

[#]Joint 1st authors

[*]To whom correspondence should be addressed:

jforwood@csu.edu.au

A

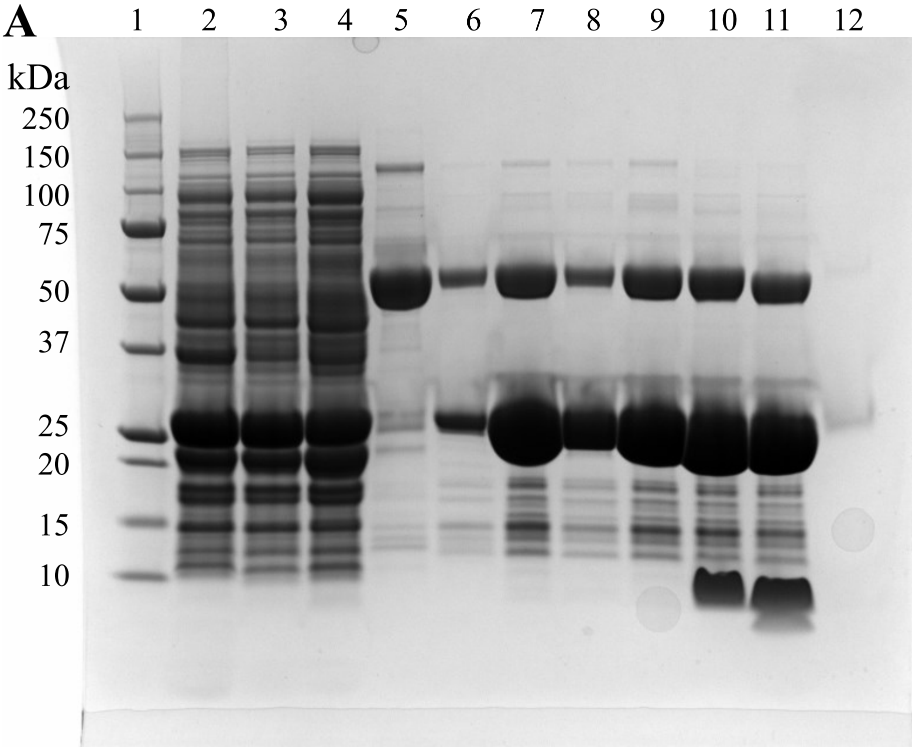

Lane	Sample
1	Molecular weight marker
2	Whole cell lysate
3	Soluble cell extract
4	GST flowthrough
5	Importin alpha injected
6	GST elution frac 1
7	GST elution frac 3
8	GST elution frac 5
9	GST pooled peak pre TEV
10	GST pooled post TEV
11	GST pooled post TEV
12	Unrelated experiment

B

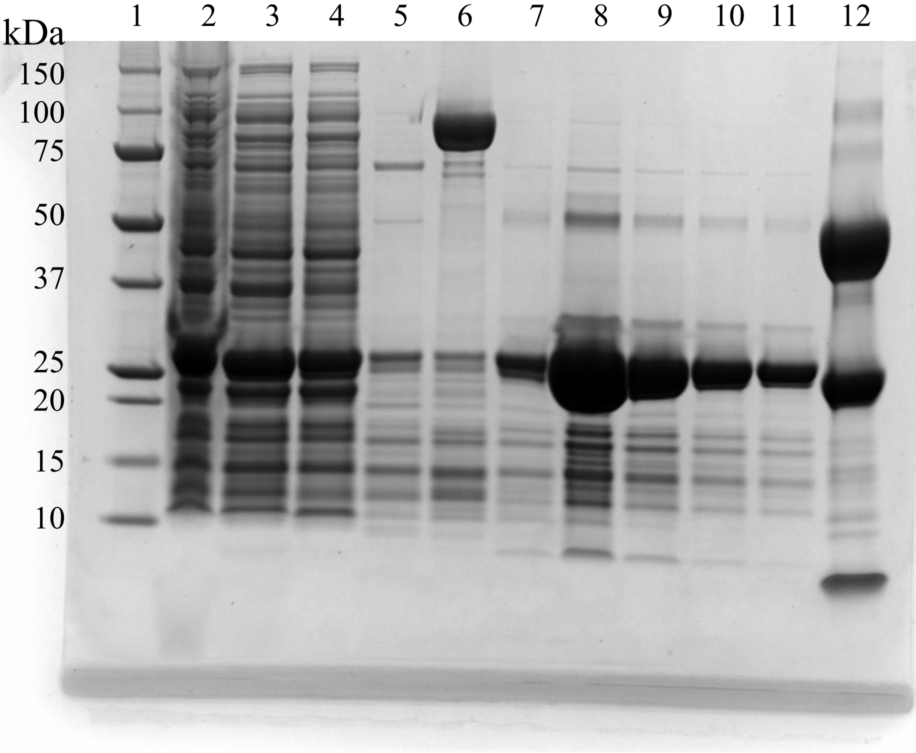

Lane	Sample
1	Molecular weight marker
2	Whole cell lysate
3	Soluble cell extract
4	GST flowthrough (frac 2)
5	Column wash
6	Importin beta injected
7	GST elution frac 1
8	GST elution frac 2
9	GST elution frac 3
10	GST elution frac 4
11	GST elution frac 5
12	Unrelated experiment

Supplementary Figure 1. Uncropped SDS-PAGE showing binding of Tat:NLS/CPP to importin-α and importin-β. A) SDS-PAGE visualization of complex formation between Tat:NLS/CPP and importin-α. B) SDS-PAGE revealing a lack of complex formation between Tat:NLS/CPP and importin-β. The cropped gels are presented in Figure 1.

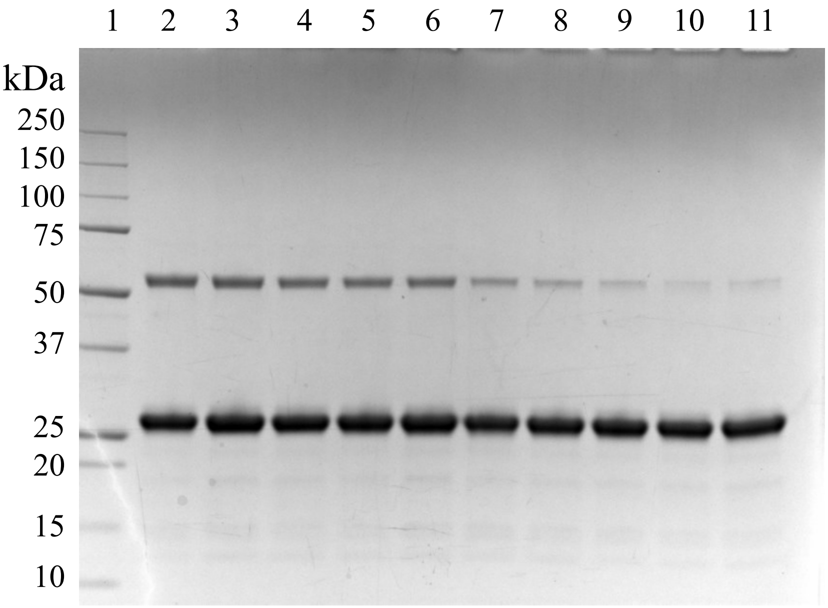

Lane	Sample
1	Molecular weight marker
2	GST-Tat Impα 30μM
3	GST-Tat Impα 15μM
4	GST-Tat Impα 7.5μM
5	GST-Tat Impα 3.75μM
6	GST-Tat Impα 1.875μM
7	GST-Tat Impα 0.938μM
8	GST-Tat Impα 0.469μM
9	GST-Tat Impα 0.234uM
10	GST-Tat Impα 0.117μM
11	GST-Tat Impα 0.059μM

Supplementary Figure 2. Uncropped gel showing quantitative GST-pull down for binding affinity determination. Glutathione agarose containing the GST-Tat:NLS/CPP incubated and washed with two-fold serially diluted importin-α (initial concentration of 30μM). Samples analysed by SDS-PAGE and images recorded using BioRad Gel Doc system were processed from triplicate gels and processed in ImageJ and analysed using one-site specific binding in Prism 7.0. The cropped gel is shown in Figure 6.
